# Supplementary material for: Charge-optimized many-body interaction potential for AlN revisited to explore plasma–surface interactions
Source: Sci Rep. 2023 Mar 31;13:5287. doi: 10.1038/s41598-023-31862-8 (PMC10066324; doi:10.1038/s41598-023-31862-8)
Supplement: Supplementary file 1 — Supplementary Information. [file 41598_2023_31862_MOESM1_ESM.pdf]

Supplementary Methods

The implementation of the LJ/ZBL tapering is verified by comparing the forces F and potential energies E of the combined LJ/ZBL with the individual LJ as well as ZBL potential for a pair of Ar atoms. F and E are shown as a function of the interatomic distance in Fig. S1 **(a)** and **(b)**, respectively.

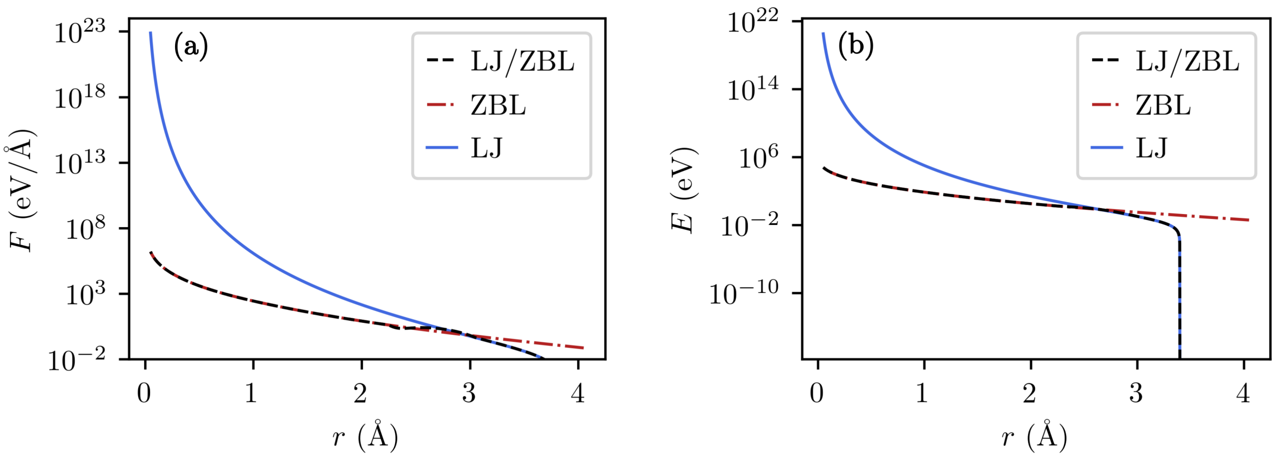

Supplementary Figure S1. **(a)** Force F between and **(b)** potential energy E of a pair of Ar atoms. Both properties are presented as a function of the interatomic distance r.

The implementation of the COMB3/ZBL tapering is verified by comparing the forces F and potential energies E of the combined COMB3/ZBL with the individual COMB3 as well as ZBL potential for a variety of cases. First, molecular nitrogen is considered. F and E are shown as a function of the interatomic distance in Fig. S2 **(a)** and **(b)**, respectively.

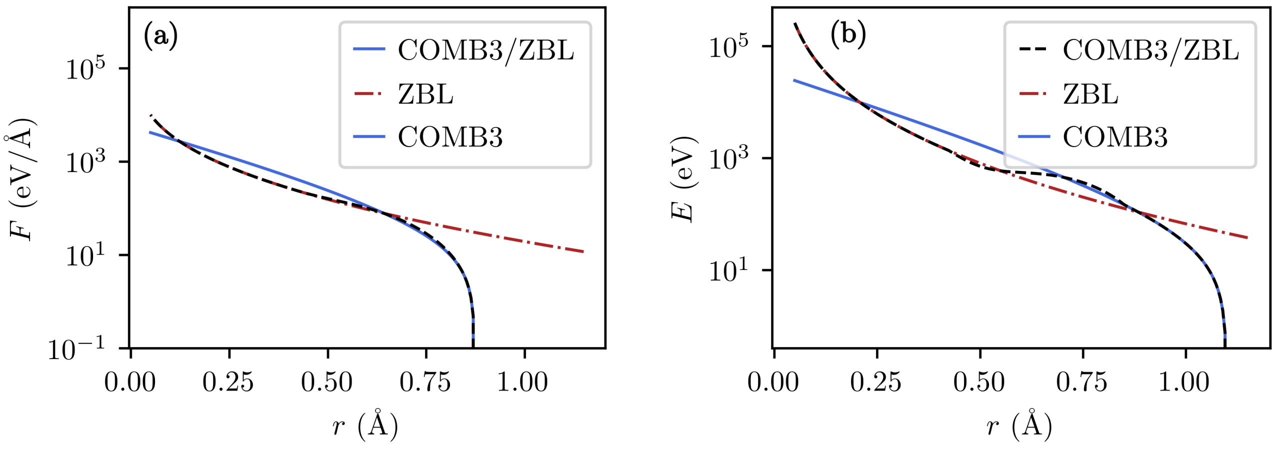

Supplementary Figure S2. **(a)** Force F between and **(b)** potential energy E of a pair of nitrogen atoms (molecular nitrogen). Both properties are presented as a function of the interatomic distance r.

Second, a N atom on top the wurtzite AlN(0001) surface is lowered until it overlaps with a N surface atom. This simulation is performed twice. In the second run, interactions between the N adatom and N surface atom are excluded. The difference of both runs are used to obtain the N-N pair interaction for this many-body scenario. F and E are shown as a function of the interatomic distance in Fig. S3 **(a)** and **(b)**, respectively.

Third, an Al atom on top the wurtzite AlN(0001) surface is lowered until it overlaps with a N surface atom. This simulation is performed twice. In the second run, interactions between the Al adatom and N surface atom are excluded. The difference of both runs are used to obtain the Al-N pair interaction for this many-body scenario. F and E are shown as a function of the interatomic distance in Fig. S4 **(a)** and **(b)**, respectively.

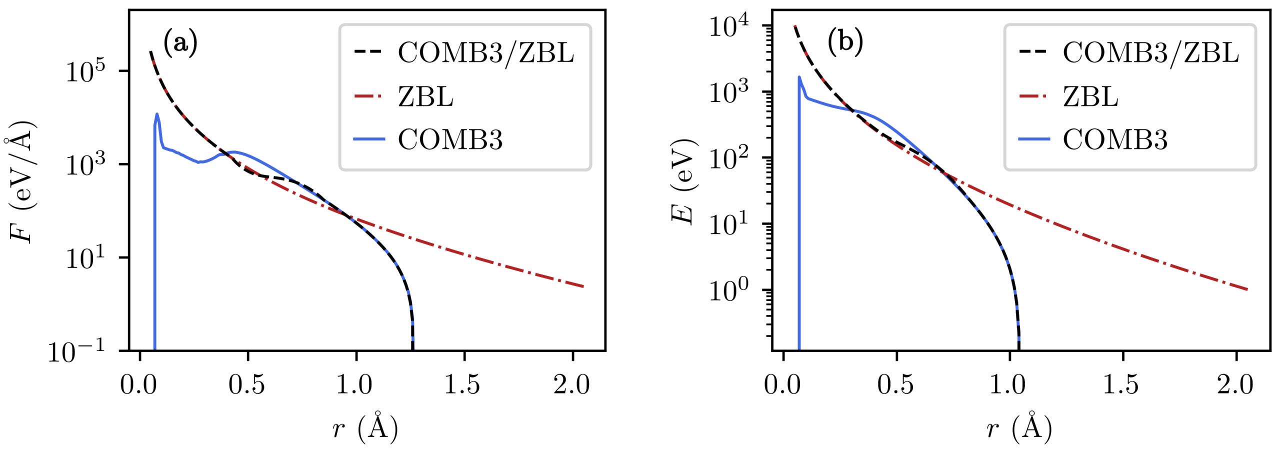

Supplementary Figure S3. **(a)** Force F between and **(b)** potential energy of a N adatom atop a N wurtzite AlN(0001) surface atom. The contribution of the remaining surface is excluded. Both properties are presented as a function of the interatomic distance r.

Fourth, an Al atom on top the wurtzite AlN(0001) surface is lowered until it overlaps with an Al surface atom. This simulation is performed twice. In the second run, interactions between the Al adatom and Al surface atom are excluded. The difference of both runs are used to obtain the Al-Al pair interaction for this many-body scenario. F and E are shown as a function of the interatomic distance in Fig. S5 **(a)** and **(b)**, respectively.

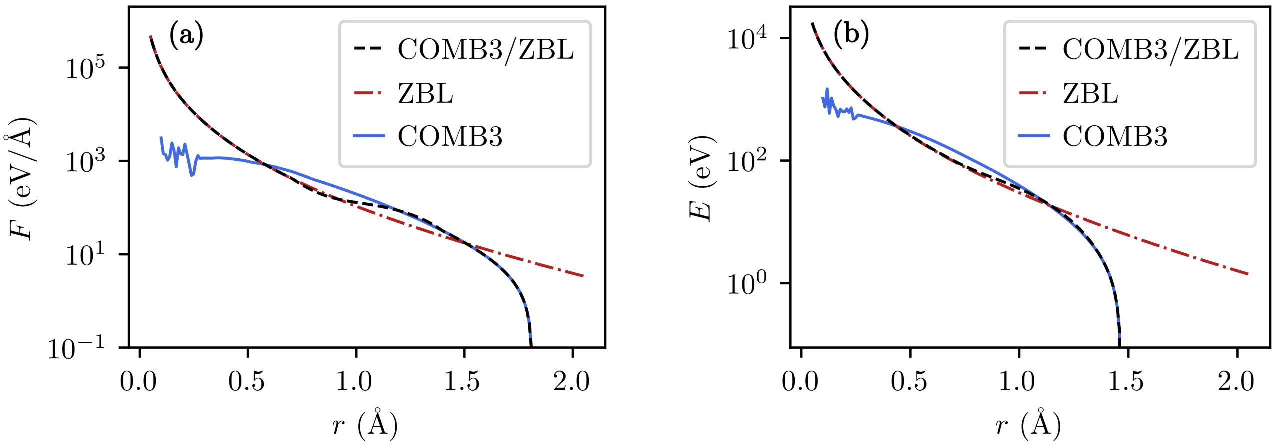

Supplementary Figure S4. **(a)** Force F between and **(b)** potential energy of an Al adatom atop an Al wurtzite AlN(0001) surface atom. The contribution of the remaining surface is excluded. Both properties are presented as a function of the interatomic distance r.

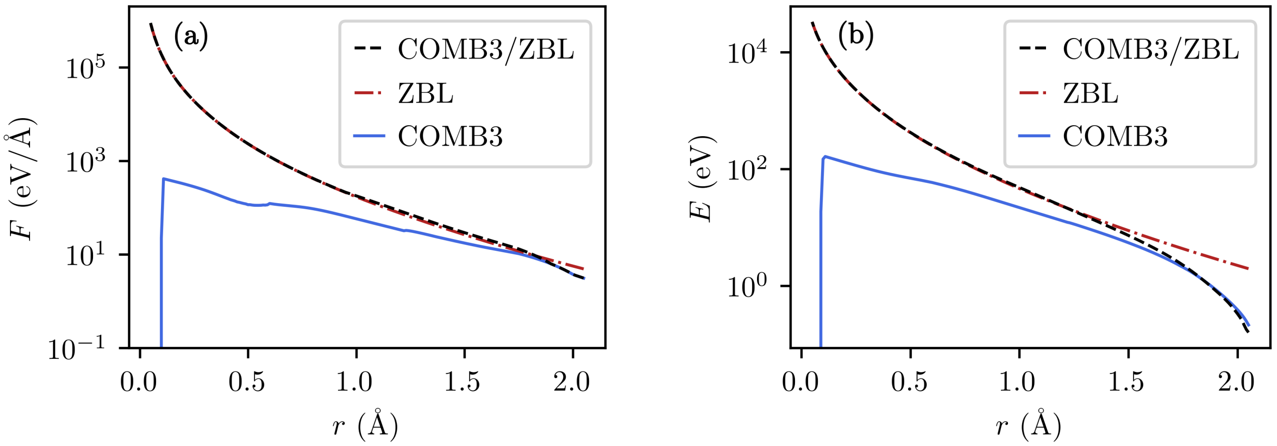

Supplementary Figure S5. **(a)** Force F between and **(b)** potential energy of an Al adatom atop an Al wurtzite AlN(0001) surface atom. The contribution of the remaining surface is excluded. Both properties are presented as a function of the interatomic distance r.

Supplementary Tables

Al_xN_y cluster structure	$COMB3^{2022}_{QTE+}$	$COMB3^{2016^*}_{QEq}$	DFT/Exp.
N_2	-4.90	-4.90	-4.88[1], -5.06[2]
Al_1N_1	-1.67	-4.37	-1.38[3], -1.39[2], -1.54[1]
Al_1N_2 (Al-N-N,C_s[4])	-3.13	-4.61	-3.52[4]
Al_1N_2 (N-Al-N,$D_{\infty h}$[4])	-1.53	-4.74	-1.80[4]
Al_1N_2 (N-Al-N,C_{2v}[4])	-3.03	-4.55	-3.51[4]
Al_2	-0.64	-0.64	-0.77[2], -0.78[1]
Al_2N_1 (Al-N-Al,C_{2v}[4])	-2.51	-3.43	-2.59[4]
Al_2N_1 (Al-Al-N,$C_{\infty v}$[4])	-1.65	-3.16	-1.41[4]
Al_2N_2 (Is[5])	-2.71	-3.33	-2.62[5]
Al_2N_2 (IIs[5])	-2.27	-3.78	-3.08[6], -3.06[5]
Al_2N_2 (IIIs[5])	-2.29	-3.13	-2.27[5]
Al_2N_2 (III[3] − V[3])	-0.51	-0.27	0.15[3]
Al_2N_2 (IV[3] − V[3])	0.92	-0.65	0.03[3]
Al_3N_2	-3.09	-2.77	-2.84[6]
Al_3N_2 (b[7] − a[7])	0.45	0.01	0.00[7]
Al_3N_3 (e[8], VIIs[5])	-2.90	-4.31	-3.28[8], -3.21[5]
Al_3N_3 (VIIIs[5])	-2.77	-4.15	-2.47[5]
Al_3N_3 (C[3] − A[3])	0.0	0.22	0.24[3]
Al_4N_2	-2.63	-2.67	-3.22[6]
Al_4N_4 (Xs[5])	-2.50	-4.16	-3.52[5]
Al_4N_4 (XIs[5])	-3.83	-3.07	-3.35[5]
Al_5N_2	-2.69	-1.95	-3.01[6]
Al_6N_2 (Al_6N_2[6])	-3.50	-2.44	-2.85[6]
Al_6N_2 (Al_6N_2-2[6])	-3.07	-2.41	-2.36[6]

Supplementary Table S1. Binding energies E_{bind} (eV/atom) of Al_xN_y cluster ($x \leq 6$, $y \leq 4$) computed with $COMB3^{2022}_{QTE+}$ and $COMB3^{2016^*}_{QEq}$ are compared with DFT and experimental findings. Labels in brackets allow for a structure identification in the provided reference when necessary. The subtraction of two labels indicates a relative binding energy.

Wurtzite	$COMB3^{2022}_{QTE+}$	$COMB3^{2016*}_{QEq}$	$COMB3^{2016}_{QEq}$	DFT/Exp.
$E_f^{V_{Al}}$ (eV)	3.63	-1.48	0.3[9]	2.36[10], 3.76[10], 4.46[10], 5.6[11]
$E_f^{Al_i^T}$ (eV)	9.14	11.10		15.83[12]
$E_f^{Al_i^O}$ (eV)	9.75	9.41		14.30[12]
$E_f^{V_N}$ (eV)	6.33	3.16	4.8[9]	5.36[10], 6.33[10], 6.46[10], 6.6[11]
$E_f^{(N\text{-}N)_N}$ (eV)	5.39	-3.51		5.05[13]
$E_f^{N_i^T}$ (eV)	5.75	-0.67		
$E_f^{N_i^O}$ (eV)	9.28	8.37		
$\gamma_{N\text{-}(0001)}$ (eV/Å^2)	0.398	0.142		0.364[14]
$\gamma_{Al\text{-}(0001)}$ (eV/Å^2)	0.127	0.102		0.136[15]
$\gamma_{(1\bar{1}00)}$ (eV/Å^2)	0.130	0.110	0.133[9]	0.145[14]
$\gamma_{(11\bar{2}0)}$ (eV/Å^2)	0.156	0.117		0.187[14]

Supplementary Table S2. Wurtzite AlN defect formation and surface energies obtained with $COMB3^{2022}_{QTE+}$ and $COMB3^{2016*}_{QEq}$ are compared to the $COMB3^{2016}_{QEq}$ AlN publication, and DFT as well as experimental findings.

Zinc blende	$COMB3^{2022}_{QTE+}$	$COMB3^{2016*}_{QEq}$	DFT/Exp.
$E_f^{V_{Al}}$ (eV)	3.32	-1.74	2.28[12]
$E_f^{Al_N}$ (eV)	11.25	7.53	15.92[12]
$E_f^{Al_i}$ (eV)	6.01	8.59	7.69[12]
$E_f^{V_N}$ (eV)	5.95	2.91	5.73[12]
$E_f^{N_{Al}}$ (eV)	5.37	5.91	5.37[12]
$E_f^{(N\text{-}N)_N}$ (eV)	5.22	-4.24	5.37[12]
$E_f^{N_i}$ (eV)	6.59	3.42	6.59[12]
$\gamma_{N\text{-}(100)}$ (eV/Å^2)	0.215	0.143	0.345[14]
$\gamma_{(110)}$ (eV/Å^2)	0.148	0.098	0.141[14]
$\gamma_{(112)}$ (eV/Å^2)	0.171	0.107	0.232[14]

Supplementary Table S3. Zinc blende AlN defect formation and surface energies obtained with $COMB3^{2022}_{QTE+}$ and $COMB3^{2016*}_{QEq}$ are compared to the $COMB3^{2016}_{QEq}$ AlN publication, and DFT as well as experimental findings.

Rock salt	$COMB3^{2022}_{QTE+}$	$COMB3^{2016*}_{QEq}$	DFT/Exp.
$\gamma_{(100)}$ (eV/Å^2)	0.130	0.170	0.083[14]
$\gamma_{(110)}$ (eV/Å^2)	0.188	0.166	0.194[14]
$\gamma_{(112)}$ (eV/Å^2)	0.171	0.097	0.270[14]

Supplementary Table S4. Rock salt AlN surface energies obtained with $COMB3^{2022}_{QTE+}$ and $COMB3^{2016*}_{QEq}$ are compared to the $COMB3^{2016}_{QEq}$ AlN publication, DFT, and experimental references.

Supplementary Results

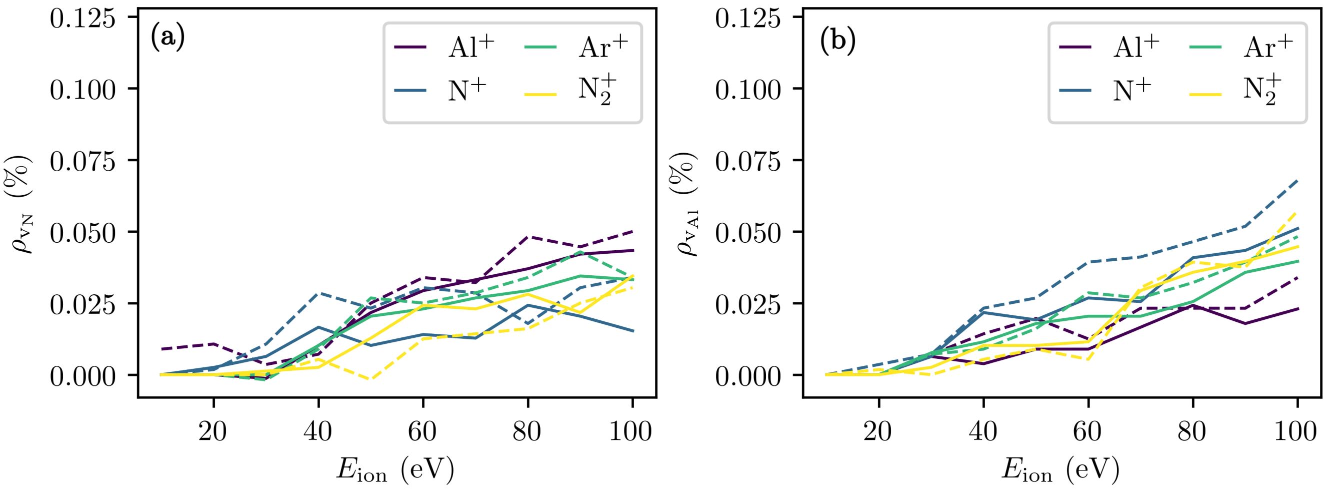

Supplementary Figure S6. **(a)** Nitrogen vacancy population ρ_{v_N} and **(b)** aluminum vacancy population $\rho_{v_{Al}}$ is presented as a function of the ion energy E and ion species (i.e., Al^+, N^+, Ar^+, N_2^+). Solid and dashed lines indicate surfaces initially equilibrated at 300 K and relaxed at 0 K, respectively.

References

1. Herzberg, G. & Huber, K.-P. *Molecular spectra and molecular structure: Infrared and Raman spectra of polyatomic molecules*, vol. 2 (Van Nostrand, Toronto; New York, 1950).
2. Nayak, S. K., Khanna, S. N. & Jena, P. Evolution of bonding in Al_nN clusters: A transition from nonmetallic to metallic character. *Phys. Rev. B* **57**, 3787–3790, DOI: 10.1103/PhysRevB.57.3787 (1998). Publisher: American Physical Society.
3. Costales, A. & Pandey, R. Density Functional Calculations of Small Anionic Clusters of Group III Nitrides. *The J. Phys. Chem. A* **107**, 191–197, DOI: 10.1021/jp022202i (2003). Publisher: American Chemical Society.
4. Kandalam, A. K. *et al.* First Principles Study of Polyatomic Clusters of AlN, GaN, and InN. 1. Structure, Stability, Vibrations, and Ionization. *The J. Phys. Chem. B* **104**, 4361–4367, DOI: 10.1021/jp994308s (2000). Publisher: American Chemical Society.
5. BelBruno, J. J. The structure of Al_nN_n (n=2–4) clusters: a DFT study. *Chem. Phys. Lett.* **313**, 795–804, DOI: 10.1016/S0009-2614(99)01073-8 (1999).
6. Costales, A., Blanco, M. A., Martín Pendás, A., Kandalam, A. K. & Pandey, R. Chemical Bonding in Group III Nitrides. *J. Am. Chem. Soc.* **124**, 4116–4123, DOI: 10.1021/ja017380o (2002). Publisher: American Chemical Society.
7. Song, B. & Cao, P.-l. Stable structures of Al_2N_3 and Al_3N_2 clusters: A full-potential LMTO molecular dynamics study. *Phys. Rev. B* **66**, 033406, DOI: 10.1103/PhysRevB.66.033406 (2002). Publisher: American Physical Society.
8. Kandalam, A. K., Blanco, M. A. & Pandey, R. Theoretical Study of Structural and Vibrational Properties of Al_3N_3, Ga_3N_3, and In_3N_3. *The J. Phys. Chem. B* **105**, 6080–6084, DOI: 10.1021/jp004404p (2001). Publisher: American Chemical Society.
9. Choudhary, K. *et al.* Dynamical properties of AlN nanostructures and heterogeneous interfaces predicted using COMB potentials. *Comput. Mater. Sci.* **113**, 80–87, DOI: 10.1016/j.commatsci.2015.11.025 (2016).
10. Hung, A., Russo, S. P., McCulloch, D. G. & Prawer, S. An ab initio study of structural properties and single vacancy defects in Wurtzite AlN. *The J. Chem. Phys.* **120**, 4890–4896, DOI: 10.1063/1.1645790 (2004). Publisher: American Institute of Physics.
11. Gorczyca, I., Svane, A. & Christensen, N. E. Theory of point defects in GaN, AlN, and BN: Relaxation and pressure effects. *Phys. Rev. B* **60**, 8147–8157, DOI: 10.1103/PhysRevB.60.8147 (1999). Publisher: American Physical Society.
12. Stampfl, C. & Van de Walle, C. G. Theoretical investigation of native defects, impurities, and complexes in aluminum nitride. *Phys. Rev. B* **65**, 155212, DOI: 10.1103/PhysRevB.65.155212 (2002). Publisher: American Physical Society.
13. Szállás, A. *et al.* Characterization of the nitrogen split interstitial defect in wurtzite aluminum nitride using density functional theory. *J. Appl. Phys.* **116**, 113702, DOI: 10.1063/1.4895843 (2014). Publisher: American Institute of Physics.
14. Holec, D. & Mayrhofer, P. H. Surface energies of AlN allotropes from first principles. *Scripta Materialia* **67**, 760–762, DOI: 10.1016/j.scriptamat.2012.07.027 (2012).
15. Jindal, V. & Shahedipour-Sandvik, F. Density functional theoretical study of surface structure and adatom kinetics for wurtzite AlN. *J. Appl. Phys.* **105**, 084902, DOI: 10.1063/1.3106164 (2009). Publisher: American Institute of Physics.
